# Modelling human lower urinary tract malformations in zebrafish

**DOI:** 10.1186/s40348-023-00156-4

**Published:** 2023-03-29

**Authors:** Caroline M. Kolvenbach, Gabriel C. Dworschak, Johanna M. Rieke, Adrian S. Woolf, Heiko Reutter, Benjamin Odermatt, Alina C. Hilger

**Affiliations:** 1 Institute of Anatomy, Medical Faculty, University of Bonn, Bonn, Germany; 2 Division of Nephrology, Department of Pediatrics, Boston Children’s Hospital, Harvard Medical School, Boston, MA USA; 3 Institute of Human Genetics, Medical Faculty, University of Bonn, Bonn, Germany; 4 Department of Neuropediatrics, University Hospital Bonn, Bonn, Germany; 5 Department of Pediatrics, Children’s Hospital Medical Center, University Hospital Bonn, Bonn, Germany; 6 Division of Cell Matrix Biology and Regenerative Medicine, School of Biological Sciences, Faculty of Biology Medicine and Health, The University of Manchester, Manchester, UK; 7 Royal Manchester Children’s Hospital, Manchester University NHS Foundation Trust, Manchester Academic Health Science Centre, Manchester, UK; 8 Division of Neonatology and Pediatric Intensive Care, Department of Pediatrics and Adolescent Medicine, Friedrich-Alexander University of Erlangen-Nürnberg, Erlangen, Germany; 9 Department of Pediatrics and Adolescent Medicine, Friedrich-Alexander University of Erlangen-Nürnberg, Erlangen, Germany; 10 Research Center On Rare Kidney Diseases (RECORD), University Hospital Erlangen, Erlangen, Germany

## Abstract

Advances in molecular biology are improving our understanding of the genetic causes underlying human congenital lower urinary tract (i.e., bladder and urethral) malformations. This has recently led to the identification of the first disease-causing variants in the gene *BNC2* for isolated lower urinary tract anatomical obstruction (LUTO), and of *WNT*3 and *SLC20A1* as genes implicated in the pathogenesis of the group of conditions called bladder-exstrophy-epispadias complex (BEEC). Implicating candidate genes from human genetic data requires evidence of their influence on lower urinary tract development and evidence of the found genetic variants’ pathogenicity. The zebrafish (*Danio rerio*) has many advantages for use as a vertebrate model organism for the lower urinary tract. Rapid reproduction with numerous offspring, comparable anatomical kidney and lower urinary tract homology, and easy genetic manipulability by Morpholino®-based *knockdown* or CRISPR/Cas editing are among its advantages. In addition, established marker staining for well-known molecules involved in urinary tract development using whole-mount in situ hybridization (WISH) and the usage of transgenic lines expressing fluorescent protein under a tissue-specific promoter allow easy visualization of phenotypic abnormalities of genetically modified zebrafish. Assays to examine the functionality of the excretory organs can also be modeled in vivo with the zebrafish. The approach of using these multiple techniques in zebrafish not only enables rapid and efficient investigation of candidate genes for lower urinary tract malformations derived from human data, but also cautiously allows transferability of causality from a non-mammalian vertebrate to humans.

**Supplementary Information:**

The online version contains supplementary material available at 10.1186/s40348-023-00156-4.

## Introduction

**Fig. 1:**
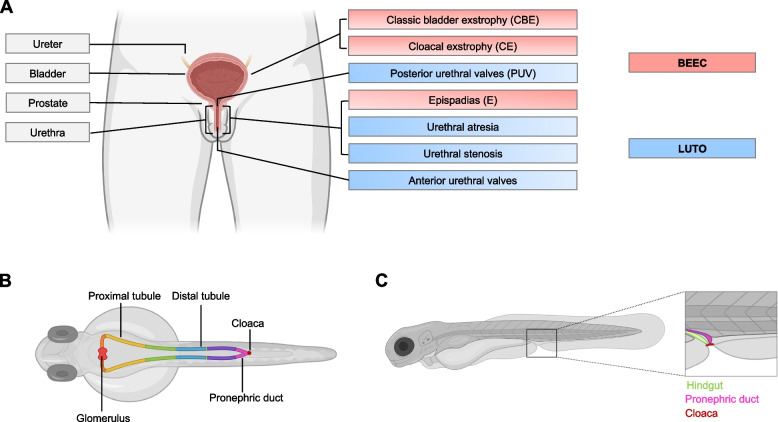
Schematic overview of human and zebrafish urinary tract. **A** Schematics of human urinary system. Topological subdivisions of the phenotypic complexity of BEEC (red) and LUTO (blue) are depicted. **B** Overview of zebrafish urinary system. It is composed of 2 nephrons with a pair of glomeruli (bright red) and tubules that can be divided in a proximal and distal segment. The proximal part is subdivided into neck (orange), proximal convoluted (yellow) and proximal straight tubule (green). The distal portion is divided in distal early (blue), corpuscle of Stannius (brown) and distal late (purple). The last segment depicts the pronephric duct (pink) that distally fuses with the cloaca (dark red). **C** Schematic depiction of cloacal region at 120 hpf. The hindgut (green) opens to the exterior, adjacent to the pronephric duct (pink), both fusing with the cloaca (dark red). Created with BioRender.com

Congenital lower urinary tract malformations (LUTMs) comprise a variety of diverse malformations affecting the bladder and urethra with a potentially severe effect on renal function in children [1, 2]. The term bladder-exstrophy-epispadias complex (BEEC; MIM 600057) refers to a spectrum of severe congenital malformations characterized by defects in the closure of the lower abdominal wall and the bladder. The severity of BEEC ranges from epispadias (E), representing the mildest form, to classic bladder exstrophy (CBE), to cloacal exstrophy (CE) (Fig. 1A) [3]. BEEC is a rare disease with an occurrence that ranges from approximately 1 in 200,000 births (CE), to 2.4 in 100,000 births (E), up to 1–2 in 50,000 births (CBE) [3]. In order to assure urinary control and to preserve renal and sexual function surgical treatment is required. Lower urinary tract obstruction (LUTO; OMIM 618612) are caused by anatomical urethral malformations, namely posterior urethral valves (PUV), anterior urethral valves, urethral stenosis, and atresia (Fig. 1A) [2]. Indeed, PUV is the most common cause of LUTO in male infants [1, 2]. Although the birth prevalence is estimated to be only 3 per 10,000 pregnancies, LUTO represent the leading cause of chronic kidney disease stage V in the first three decades of life [4, 5]. Moreover, the mortality with PUV is reported to be as high as 45% [5]. Functional rather than anatomical bladder or urethral outflow obstruction occurs in, e.g., urofacial syndrome and some cases of prune belly syndrome.

The cause of anatomical LUTMs remains unknown in the majority of cases. Nevertheless, there is evidence that some of the before mentioned malformations may occur due to defective cloacal development. PUV are thought to be the outcome of an abnormal insertion of the Wolffian duct into the cloaca, thus causing abnormal ridges in the posterior urethra [6]. BEEC is caused by a developmental abnormality of the cloacal membrane, which is not replaced by tissue that will form the abdominal muscles [1, 3, 7, 8]. However, it is assumed that due to the heterogeneity of the BEEC spectrum several organ fields may be involved, and CE is sometimes considered to have a different embryologic origin from CBE [3, 8].

Occurrence of familial cases (i.e., a positive family history of disease) that appear to follow a Mendelian mode of inheritance and the fact that the development of the urinary tract is governed by distinct developmental genes support the hypothesis that genetic factors play an important role in the formation of these malformations [9–13]. For example, *WNT3* and *WNT9B*, regulators of the canonical Wnt/beta-catenin signaling pathway, have been shown to play a leading role during bladder development [7, 14]. Furthermore, *Wnt3* and *Wnt9b* were shown to be expressed in the developing mouse genital tubercle which corresponds to the period of bladder development in humans [7]. Molecular tools such as exome sequencing allow the identification of potential novel candidate genes causing congenital LUTMs and have improved our understanding of underlying disease mechanisms. Recently, we reported heterozygous variants in *BNC2*, encoding a zinc finger transcription factor potentially involved in nuclear mRNA processing, as the first disease gene for isolated anatomical LUTO [15, 16]. Furthermore, variants in *WNT3* and *SLC20A1*, a sodium-phosphate symporter, have been shown to be implicated in the formation of BEEC [14, 17]. Further characterization of candidate gene function and proving causality of the genetic variants identified can be based on various animal models. Although mouse models have significant advantages, they are limited in use for fast large-scale studies including genetic manipulation. The zebrafish (*Danio rerio*) complements these deficits. Here, we highlight the use of the zebrafish model for studying anatomical LUTMs.

### Zebrafish—a suitable model for LUTMs

**Fig. 2:**
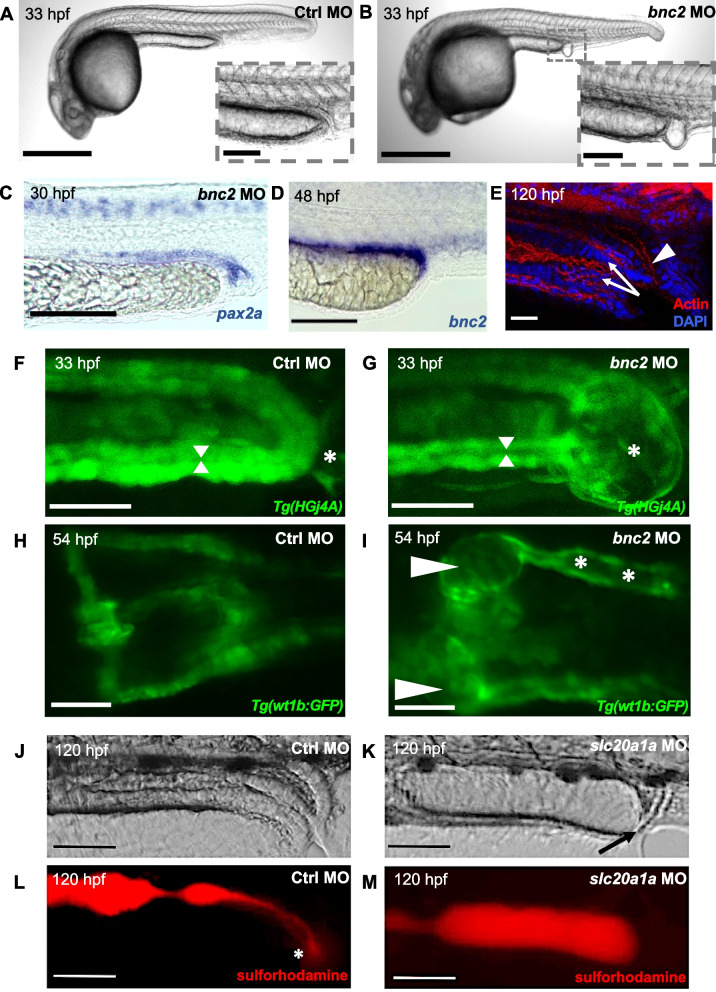
Zebrafish as a model for developmental lower urinary tract defects. **A**, **B** Zebrafish injected with *bnc2* Morpholino (MO) frequently develop a pronephric distal outlet obstruction (‘vesicle’; enlargement in **B**) at 33 hpf compared with controls. **C** WISH with a *pax2a* probe relates the *bnc2* MO induced pronephric outlet obstruction to distal parts of the pronephric ducts and the cloaca. **D** At 48 hpf *bnc2* was expressed in the terminal section of the pronephric ducts. **E** Staining for actin (red) and DAPI (blue) allows the visualization of the hindgut (white arrow) and distal pronephric duct (white arrow head) in wild-type zebrafish larvae. **F**, **G ***Tg(HGj4A)* zfl in dorsal view at 33 hpf showing distal pronephric outlet obstruction (asterix) with resulting dilatation of pronephric ducts (indicated by arrow heads) in *bnc2* MO zfl (**G**) compared to zfl injected with control (Ctrl) MO. **H**, **I** Proximal pronephric region of *Tg(wt1b:GFP)* in dorsal view with cystic dilation of glomeruli (arrow heads) and pronephric ducts (asterisks) in *bnc2* MO zfl, which recapitulate human hydronephrosis (**I**). **J**–**M** Zfl are depicted after the ingestion of sulforhodamine. Pictures are shown in brightfield (**J**, **K**) and fluorescent red channel (**L**, **M**). The intestine and cloaca appear regular and ensure excretion of the red dye (asterisk in **L**) in controls. *slc20a1*a *knockdown* impairs excretory function of the cloaca (black arrow in **K**) and leads to distension of the hindgut. Scale bars: **A**, **B** 500 μm (100 μm magnification in **B**), **C**, **D**; **F**–**I** 100 μm, **E** 30 μm, **J**–**M**: 50 μm. **A**–**C**; **F**–**I** Modified from Kolvenbach et al. [15]. **J**–**M** Modified from Rieke et al. [17]

The zebrafish has emerged as a promising non-mammalian vertebrate model for studying LUTMs. Key advantages include large numbers of offspring with rapid *ex utero* development and anatomic structures with similarity to the human developing urinary tract. The embryonic kidney, also called pronephros, forms by patterning of intermediate mesoderm [15, 16]. The anterior intermediate mesoderm develops into two filtering glomeruli fused at the midline (Fig. 1B) [15, 16]. Each is linked to a pronephric tubule, connecting the glomeruli to the pronephric ducts, that distally fuse at the cloaca opening, forming around 24–30 h post-fertilization (hpf) (Fig. 1B-C). At 48 hpf, the hindgut also inserts into the cloaca, which only opens for excretion at around 120 hpf (Fig. 1C) [15, 16]. Zebrafish do not have a urinary bladder for urine storage, but similar anatomic excretion surrogates, such as the distal pronephric ducts and cloaca, that allow the analysis of the developing lower urinary tract (Fig. 1B-C) [14, 18]. For instance, the terminal end of the pronephros represents a putative homolog to the human Wolffian duct that inserts into the bladder [14, 18]. Similarly, the cloaca ensures urine outflow corresponding to the human urethra. Defects of the cloaca have been previously successfully modeled in zebrafish [19–22]. BEEC and LUTO likely arise from abnormal development of the cloaca, making zebrafish a suitable model for analyzing pathomechanisms underlying the diseases. Another key advantage of zebrafish includes easy and fast genetic modulation. There is evidence for extensive evolutionary conservation between human and zebrafish genomes with 84% of human genes associated with human disease having a zebrafish ortholog [23]. Similarly, *Drosophila* (*D*.) *melanogaster* and *Caenorhabditis* (*C*.) *elegans*, two other valuable non-mammalian model organisms are also characterized by simple genetic manipulation, but only share 77% (*D. melanogaster*) [24] and 65% (*C. elegans*) [25] of matching disease genes. Modeling of human genetic disease in zebrafish can be achieved by using Morpholino®-based *knockdown* and CRISPR/Cas genome editing. The term Morpholino refers to an oligomer molecule, inhibiting formation of the translation-initiation or splicing complex, thereby *knocking down* gene function [26]. CRISPR/Cas uses guide RNA that recognizes complementary DNA sequences, signaling the Cas9 to cut the double-stranded DNA at the targeted location [27]. Non-homologous end joining cellular repair mechanisms introduce insertions or deletions, resulting in gene *knock-out* through genomic disruption [27]. Previous studies have shown the utility of transient suppression models or stable mutants for testing disease relevance in a very short time period as compared with mammalian vertebrate models [15, 28]. In our recent study, we showed that CRISPR/Cas F0 mosaic mutants or Morpholino *knockdown* of *bnc2* in zebrafish results in a distal pronephric outlet obstruction, illustrated as a ‘vesicle’, phenocopying the human anatomical LUTO phenotype (Fig. 2A-B) [15]. Furthermore, Morpholino-based *knockdown* of the zebrafish orthologs *wnt3* and *slc20a1a* caused expansion of the cloacal lumen, implicating their important role in urinary tract development and potential involvement in BEEC formation [14, 17]. In addition, we used the zebrafish model to determine the pathogenicity of variants identified in our LUTO cases with variants in *BNC2* [15]. Together with *bnc2* Morpholino, *knocking down* endogenous *bnc2* zebrafish RNA, human *BNC2* RNA bearing identified variants was co-injected into zebrafish embryos in one-cell stage. Human *BNC2* RNA lacks the Morpholino-binding site and is thereby not detected by the Morpholino. No amelioration of the described LUTO-like phenotype was observed in contrast to co-injections of wild-type human *BNC2* RNA together with *bnc2* Morpholino [15]. The pathogenicity of the tested variants can therefore be assumed. Alternatively, patient-specific variants can be introduced applying CRISPR/Cas9 (*knock-in*) requiring a donor template for homology-directed repair in addition to the Cas9 enzyme and guide RNA [29]. The emergence of a phenotype corroborates the pathogenicity of the targeted variant.

### Assessment of urinary tract phenotypes

Zebrafish larvae (zfl) are nearly transparent, which allows direct examination and visualization of developing structures under the microscope. Their transparency also facilitates staining of designated tissue. Whole-mount in situ hybridization (WISH) using RNA probes to detect the mRNA of interest, can be applied for locating gene expression [30]. The transcription factors *pax2a* and *evx1* are established WISH marker molecules that highlight the developing distal pronephric and cloacal component structures, responsible for urine excretion [22]. Abnormalities during the cloaca-forming process can thus be easily detected using WISH in zebrafish. Staining against *pax2a* in our *bnc2 knockdown* zebrafish indicated the intimate relation of the ‘vesicle’ with the distal pronephric duct at the cloacal opening (Fig. 2C) [15]. Moreover, WISH serves as a powerful tool for studying the expression of potential new disease genes. Specific probes for the gene of interest can be easily invoked to depict location-specific expression at the critical time points of urinary tract development. In this manner, the expression of *bnc2* was detected in developing cloacal and pronephric duct tissue (Fig. 2D) [15, 17]. Similarly, phalloidin staining can be employed to visualize cytoskeleton actin filaments and by this to analyze cloaca morphogenesis (Fig. 2E) [14, 19, 31]. This allows the evaluation of cellular organization of the cloacal epithelium and size of the lumen of the distal pronephros and hindgut inserting into the cloaca (Fig. 2E). Using this method, Baraknowska Korberg et al. [14] detected cloacal expansion and cellular disorganization potentially arising from cloacal obstruction in *wnt3 knockdown* zebrafish morphants. Phenotypic anomalies can be simply visualized with transgenic lines expressing fluorescent protein under a tissue-specific promoter in the nearly transparent zfl. For example, the mnr2b/hlxb9lb enhancer trap line *Tg(HGj4A)* expressing green fluorescent protein (GFP) in the developing distal pronephric region has been used to illustrate the distal pronephric outlet obstruction in vivo after *bnc2* Morpholino knockdown (Fig. 2F–G) [15, 32]. Furthermore, *Tg(wt1b:GFP)* zfl harboring GFP under a Wilms Tumor 1b gene promoter can be conveniently screened for renal and tubular dilatations [33], which occurred in genetic models for *bnc2* and *slc20a1a,* strongly supporting the hypothesis that the observed cloacal malformations lead to a blockage of urine outflow (Fig. 2H-I) [15, 17]. This observed upper urinary tract phenotype in zebrafish might resemble hydronephrosis seen in individuals with LUTMs due to obstruction.

As a useful functional assessment to determine the in vivo functionality of the hindgut and cloaca, a sulforhodamine excretion-assay can be applied [17, 31]. Here, zfl are bathed in sulforhodamine, a red fluorescent dye, for several hours at day 5 post-fertilization. They ingest and excrete it in intervals, allowing to observe normal or abnormal cloacal physiology (Fig. 2J–M). In the *slc20a1a knockdown* zebrafish model for BEEC, normal morphology of the intestine and regular peristalsis was observed, but cloacal opening seemed to be impaired shown by dilation of the hindgut and cloaca (Fig. 2J–M) [17].

The benefits of the zebrafish model have also been demonstrated in the evaluation of candidate genes for upper urinary tract abnormalities. For example, Brophy et al. [34] and Sanna-Cherchi et al. [35] showed that *greb1l*, the human ortholog *GREB1L* being a candidate disease gene for renal hypoplasia, is required for normal pronephros morphogenesis in zebrafish. Suppression of *greb1l* resulted in proximal pronephric defects, recapitulating the human phenotype. Again, this model was also successfully used, to determine the pathogenicity of discovered human missense variants, making the zebrafish a suitable model for congenital kidney and upper urinary tract malformations.

### Limitations of the zebrafish model

Zebrafish lack human anatomic structures such as genitalia and urinary bladder, making it a potentially distant model for a subset of human urinary tract phenotypes. Nevertheless, they have been successfully used to model several human anatomical urinary tract malformations, phenocopying human anomalies caused by genetic alterations [14, 15, 17]. However, modelling functional LUTO probably requires a mammalian model with the muscularized/innervated bladder. Other limitations may include the lack of expression of a candidate gene in the urinary tract of the zebrafish, requiring its characterization in a mammalian model. However, the zebrafish lower urinary tract, namely the distal pronephric ducts and cloaca, provides a powerful, model system to test a genetic hypothesis generated for certain candidate genes from human data so far. The approach of using these multiple techniques does not only enable rapid and efficient investigation of candidate genes for LUTMs, but also cautiously allows transferability of causality from the non-mammalian vertebrate zebrafish model to humans.

## Supplementary Information

**Additional file 1.**

## Data Availability

The datasets used and/or analyzed during the current study are available from the corresponding author on reasonable request.

## Abbreviations

BEEC: Bladder-exstrophy-epispadias complex
CBE: Classic bladder exstrophy
CE: Cloacal exstrophy
CRISPR/Cas: Clustered regularly interspaced short palindromic repeats/CRISPR-associated protein 9
Ctrl: Control
E: Epispadias
GFP: Green fluorescent protein
hpf: Hours post-fertilization
LUTM: Lower urinary tract malformation
LUTO: Lower urinary tract obstruction
MO: Morpholino
PUV: Posterior urethral valves
Tg: Transgenic
WISH: Whole-mount in situ hybridization
Zfl: Zebrafish larvae
