# Supplementary material for: Modelling human lower urinary tract malformations in zebrafish
Source: Mol Cell Pediatr. 2023 Mar 29;10:2. doi: 10.1186/s40348-023-00156-4 (PMC10050536; doi:10.1186/s40348-023-00156-4)
Supplement: Supplementary file 1 — Additional file 1. [file 40348_2023_156_MOESM1_ESM.docx]

**SUPPLEMENT**

**METHODS**

**Phalloidin stainings**

Phalloidin stainings were performed as reported previously[1-3]. Zebrafish larvae (zfl) were fixed at 5 days post fertilization (dpf) in 4% paraformaldehyde (PFA) overnight at 4°C. After fixation zfl were washed three times for 5 minutes in phosphate-buffered saline (PBS) with 0.1% Tween-20 (PBST) followed by 5 minutes in PBS with 2% Triton X-100 (PBSTx). Subsequently, zfl were incubated in PBSTx for 2 hours to ensure permeabilization. Stainings were conducted overnight at 4°C with tetramethyl rhodamine B isothiocyanate (2ug/mL; Sigma Aldrich P1951). Zfl were then incubated in DAPI (ACDBio, RNAscope DAPI, 320858) overnight at 4°C before imaging.

**Whole-mount *in situ* hybridization (WISH)**

Probe synthesis was performed as previously described[1, 4]. Antisense probes for *bnc2* were transcribed from cDNA clone IMAGE:7063815 (Source BioScience) and linearized by restriction enzymes. Dig-labelled RNA was generated by using Roche Dig labelling kit. WISH was then performed according to Thisse *et al.*[5]

**Imaging**

Selected zfl were mounted in 2% low-melting agarose and analyzed by a Nikon AZ100 Macro-Zoom microscope. Phalloidin-stained zfl were imaged by a two-photon scanning fluorescence microscopy (LaVision Trim-Scope II; ImSpector and ImageJ software)[1, 6].

**REFERENCES**

1. Kolvenbach CM, Felger T, Schierbaum L, Thiffault I, Pastinen T, Szczepanska M, et al. X-linked variations in SHROOM4 are implicated in congenital anomalies of the urinary tract and the anorectal, cardiovascular and central nervous systems. J Med Genet. 2022. doi: 10.1136/jmg-2022-108738.

2. Parkin CA, Allen CE, Ingham PW. Hedgehog signalling is required for cloacal development in the zebrafish embryo. Int J Dev Biol. 2009;53(1):45-57. doi: 10.1387/ijdb.082669cp.

3. Baranowska Korberg I, Hofmeister W, Markljung E, Cao J, Nilsson D, Ludwig M, et al. WNT3 involvement in human bladder exstrophy and cloaca development in zebrafish. Hum Mol Genet. 2015;24(18):5069-78. doi: 10.1093/hmg/ddv225.

4. Kolvenbach CM, Dworschak GC, Frese S, Japp AS, Schuster P, Wenzlitschke N, et al. Rare Variants in BNC2 Are Implicated in Autosomal-Dominant Congenital Lower Urinary-Tract Obstruction. Am J Hum Genet. 2019;104(5):994-1006. doi: 10.1016/j.ajhg.2019.03.023.

5. Thisse C, Thisse B. High-resolution in situ hybridization to whole-mount zebrafish embryos. Nat Protoc. 2008;3(1):59-69. doi: 10.1038/nprot.2007.514.

6. Rieke JM, Zhang R, Braun D, Yilmaz O, Japp AS, Lopes FM, et al. SLC20A1 Is Involved in Urinary Tract and Urorectal Development. Front Cell Dev Biol. 2020;8:567. doi: 10.3389/fcell.2020.00567.
